# Update in the clinical utilization of chemoprevention for breast cancer: a narrative review

**DOI:** 10.3389/fonc.2025.1435253

**Published:** 2025-06-17

**Authors:** Razan Mansour, Mahmoud Abunasser, Baha’ Sharaf, Hikmat Abdel-Razeq

**Affiliations:** ^1^ Department of Internal Medicine, University of Kansas Medical Center, Kansas City, KS, United States; ^2^ Department of Internal Medicine, Section of Hematology and Medical Oncology, King Hussein Cancer Center, Amman, Jordan; ^3^ School of Medicine, The University of Jordan, Amman, Jordan

**Keywords:** chemoprevention, breast cancer, tamoxifen, aromatase inhibitors, AI, exemestane, anastrozole

## Abstract

**Background:**

Breast cancer, a leading cause of cancer-related deaths, prompts research into chemoprevention strategies. This narrative review explores risk factors, assessment tools, and summarizes the effectiveness and side effects of chemopreventive agents used for breast cancer risk reduction;

**Methods:**

Published data from major clinical trials, meta-analyses, and data presented at major international conferences that addressed the utilization of tamoxifen, raloxifene, aromatase inhibitors (AI) and other potential drugs are reviewed. Risk assessments models utilized to assess women’s risk of getting breast cancer are discussed, too;

**Results:**

Tamoxifen, a selective estrogen receptor modulator (SERM), demonstrated efficacy in reducing breast cancer risk in postmenopausal and premenopausal women. However, it poses several worrisome adverse events. Raloxifene, another SERM, has risk-reducing benefits with a better safety profile compared to tamoxifen. AI, like anastrozole and exemestane, reduced invasive breast cancer with better side effect profile. Denosumab, a monoclonal antibody that tackles receptor activator of nuclear factor kappa B (RANK-RANKL), is promising in preventing breast cancer in healthy carriers of pathogenic *BRCA1* variants. Despite their proven efficacy, chemopreventive agents are underutilized underscoring the importance of raising the awareness of health care workers and women at-risk;

**Conclusion:**

Chemopreventive agents present opportunities for reducing breast cancer risk. However, careful consideration of side effects and individual risk factors are crucial to enhance uptake rate. Further research is needed to compare the effectiveness of SERMs and AI in preventing breast cancer, especially in high-risk populations with pathogenic germline mutations.

## 1 Introduction

Breast cancer is the most prevalent non-skin cancer in women and is considered the second leading cause of cancer-related mortality (1, 2). Women are increasingly susceptible to breast cancer; since 2008, the annual growth rate in newly diagnosed cases has reached 20%. With 2.3 million reported new cases in 2020, and approximately 685,000 deaths annually, it stands as the most prevalent form of cancer (3). Multiple risk factors were reported to contribute to the development of breast cancer. Age remains a significant determinant, with the risk increasing steadily with advancing age. Approximately 80% of breast cancer cases are diagnosed in women aged 50 and older (4). Hormonal factors, such as early menarche, late menopause, and nulliparity, play a crucial role by contributing to alterations in estrogen levels (5–7). Lifestyle factors also contribute significantly; a meta-analysis by Key et al. estimated that approximately 30% of breast cancer cases could be prevented through lifestyle modifications, including maintaining a healthy weight, regular physical activity, and limiting alcohol consumption (8).

In addition to the above risk factors, genetic predispositions significantly contribute to the etiology of breast cancer. BRCA mutations, specifically those affecting the *BRCA1* and *BRCA2* genes, play a pivotal role as tumor suppressors involved in DNA repair processes. Mutations in *BRCA1* and *BRCA2* genes disrupt these repair mechanisms, predisposing individuals to an elevated risk of breast and ovarian cancers (9–11). *BRCA1* mutation carriers have a lifetime risk of approximately 60-70% for developing breast cancer, while those with *BRCA2* mutations have a risk of around 45-55%. Inherited breast cancer contributes to 10% or more of all newly diagnosed breast cancer (12–14).

Chemoprevention has gained growing attention in the field of breast cancer as an approach to reduce cancer risk, and multiple studies have explored the impact of chemopreventive drugs on decreasing the incidence of breast cancer (15–17). Three key agents, tamoxifen, raloxifene, and aromatase inhibitors (AI), stand out as risk reduction agents in patients at high risk for breast cancer (18). The choice of a specific chemopreventive agent involves a careful assessment of an individual’s risk factors, potential side effects, and patient’s preferences. This narrative review provides a snapshot and summarizes the current landscape of chemoprevention in breast cancer, to provide an understanding of the impact of chemopreventive interventions on breast cancer incidence.

## 2 Risk assessment tools

Several risk assessment tools are commonly used to estimate breast cancer risk based on various risk factors.

### 2.1 The Gail model

Gail et al. created the Gail model to estimate breast cancer risk based on risk factors (19). The model considers a woman’s birth age, race/ethnicity, age at menarche, history of breast biopsy and current age. This data allows the Gail model to estimate absolute 5-year, 10-year, and lifetime breast cancer risk. The 1989 Gail model for breast cancer risk assessment, prevention, and screening has grown into the new Gail model, also known as the Breast Cancer Risk Assessment Tool 2.0, which was updated to improve risk stratification. The new model expands on family history and biopsy history, considering the number of previous breast biopsies, presence of atypical hyperplasia, and number of woman’s first-degree relatives with breast cancer (20–23). While the Gail Model is a valuable tool, it has some limitations, such as not incorporating certain risk factors like breast density or genetic mutations. Additionally, it is more applicable to the general population rather than specific high-risk ethnic groups (24).

### 2.2 Breast Cancer Surveillance Consortium risk calculator

The interactive Breast Cancer Surveillance Consortium (BCSC) model estimates a woman’s six-year breast cancer risk using data from annual or biennial screening mammograms (25, 26). The BCSC tool uses risk factors like age, ethnicity, family history, and breast density to provide a personalized risk assessment based on a woman’s individual characteristics and screening history. The BSBC model is particularly useful for assessing and treating dense breasts on mammography. Using breast density and other risk factors from mammogram screening, the BCSC estimates personalized breast cancer risk for screening and prevention.

### 2.3 Tyrer-Cuzick (IBIS) model

In addition to age, breast density, family history, and menopausal status, the Tyrer-Cuzick (IBIS) Model incorporates the use of hormone replacement therapy (HRT), including duration and recency, into its’ risk stratification. The model distinguishes between proliferative breast disease with and without atypia, recognizing that these conditions may confer different levels of risk (27).

### 2.4 Genetic risk assessment models

The BOADICEA (Breast and Ovarian Analysis of Disease Incidence and Carrier Estimation Algorithm) model is a breast cancer risk assessment tool that integrates both family history and genetic information for cancer risk assessment. It evaluates the risk of breast and ovarian cancers by considering data on multiple family members, ages at diagnosis, and the presence of specific genetic mutations, including *BRCA1, BRCA2, TP53*, and others (28). The BRCAPRO model is a similar risk assessment tool that primarily focuses on the assessment of risk associated with *BRCA1* and *BRCA2* mutations. It also provides estimates of the probability of carrying a BRCA mutation (29). While both tools provide input on genetic risk assessment, BOADICEA integrates detailed family history data, providing a more comprehensive evaluation by considering specific cancer types within the family.

While each model has its unique strengths and applications, they share a common goal of assessing breast cancer risk to guide personalized prevention and screening strategies. The Gail Model is widely used for general risk assessment, while the IBIS model incorporates additional factors for postmenopausal women. The BCSC Risk Calculator, utilizing real-world data, offers a personalized approach based on individual characteristics and screening history. Models like BRCAPRO and BOADICEA are specifically tailored for individuals with a family history of breast and ovarian cancers, providing valuable insights into genetic mutations, including *BRCA1* and *BRCA2*, as primary risk factors.

The United States Preventive Services Task Force (USPSTF) evaluated the accuracy of risk assessment models to identify women who could benefit from primary breast cancer chemoprevention (30, 31). The evidence profile included 25 studies and 18 risk assessment models (n > 5 million participants). Most included studies reported AUC (area under the curve) values from 0.55 to 0.65, indicating low accuracy in predicting incidence of breast cancer in individual women. One study reported AUC > 0.70 for both the Tyrer-Cuzick and the Gail-2 models.

These risk assessment models were implemented in real-world practice. Guidelines from professional groups including the American Society of Clinical Oncology (ASCO), National Comprehensive Cancer Network (NCCN), and the USPSTF recommend counselling women over 35 years with high breast cancer risk (32–34). The high-risk thresholds for inclusion in the guidelines were similar between the three groups but varied slightly. According to ASCO, high-risk criteria warranting preventive action included women aged 35 years or older, with a minimum 10-year life expectancy, and has one of the following: a past history of receiving radiation therapy targeting the thoracic region before getting to thirty years old, a history of *in situ* lobular carcinoma (LCIS), five-year risk of breast cancer of ≥1.7% (found, for instance, on the modified Gail model), or atypical ductal hyperplasia (ADH) on biopsy.

## 3 Risk reduction agents

### 3.1 Tamoxifen

Tamoxifen has been used to treat early and advanced breast cancer for more than three decades (35). The National Surgical Adjuvant Breast and Bowel Project (NSABP) P-1 trial randomized 13,388 pre- and post-menopausal women to receive either a placebo or tamoxifen (20 mg/day) for five years (32). The incidence of invasive breast cancer decreased from 42.5 per 1000 women in the placebo group to 24.8 per 1000 women in the tamoxifen group (RR 0.57, 95% CI 0.46 to 0.70). The risk reduction associated with tamoxifen was evident across all eligible age groups and was observed in women with a history of lobular carcinoma *in situ* (LCIS) (RR 0.44; 95% CI 0.16 to1.06) and those with a history of atypical lobular or ductal hyperplasia (RR 0.14; 95% CI 0.03 to0.47). However, the study also reported a notably increased risk of thromboembolic events and endometrial cancer in the tamoxifen group compared to the placebo group. These risks were higher in women aged 50 years and older, with the incidence of thromboembolic events particularly elevated within the first three years of tamoxifen use. Two studies were published after the P-1 trial, the Royal Marsden Hospital Tamoxifen Prevention Pilot Trial (33) and the Italian Tamoxifen Prevention Study (34).

In a randomized controlled trial (IBIS-I) conducted by Cuzick et al. (36), postmenopausal and premenopausal women were randomized to receive oral tamoxifen at a dosage of 20 mg/day versus placebo, spanning a duration of five years. The trial, conducted between 1992 and 2001, involved a total of 7154 women, with 3575 participants assigned to the placebo group and the remaining 3579 to the tamoxifen group. Over a 16-year follow-up period, breast cancer was diagnosed in 251 (7.0%) women among women who received tamoxifen, compared to 350 (9.8%) cases observed in the placebo group (hazard ratio [HR] 0.71 95% CI 0·60 to 0·83, p < 0.0001). Overall, there was no significant difference in all-cause mortality between treatment groups (odds ratio [OR] 1.10, 95% CI 0·88 to 1·37, p= 0.4).

The Royal Marsden study involved 2494 healthy women and was conducted over an 8-year period, randomized a placebo and oral tamoxifen (20 mg/day) regimen to evaluate the efficacy of the medication in preventing breast cancer (33). The primary endpoint of the study was the occurrence of breast cancer, with 1233 patients assigned to the placebo arm and 1238 to the tamoxifen arm. The overall results revealed that 104 participants in the placebo group developed breast cancer, compared to 82 individuals in the tamoxifen group at a median of 13-year follow up (HR 0.78, 95% CI = 0.58 to 1.04; p= 0.1). However, the study observed a statistically significant reduction in the incidence of ER-positive breast cancer in the tamoxifen arm (HR 0.61, 95% CI = 0.43 to 0.86; p= 0.005).

One of the earlier trials to explore tamoxifen use in postmenopausal women was the Italian Tamoxifen Prevention Study, led by Veronesi et al. The trial aimed to investigate the preventive potential of tamoxifen in postmenopausal women who had undergone hysterectomy. Participants were randomly assigned to receive either tamoxifen at a dose of 20 mg daily or a placebo orally for a planned duration of 5 years. There was no difference in breast-cancer frequency between the placebo (n= 22) and tamoxifen (n= 19) arms. A statistically significant reduction in breast cancer incidence was observed among women receiving tamoxifen who concurrently used hormone-replacement therapy during the trial. In comparison to the placebo group, women on tamoxifen exhibited a significantly elevated risk of vascular events and hypertriglyceridemia [34}.

### 3.2 Mini-dose tamoxifen

Given the frequent and potentially disturbing adverse events associated with tamoxifen, especially when used in postmenopausal women, researchers have explored the use of lower doses of tamoxifen (mini-dose tamoxifen). A phase-III trial studied the role of mini-dose tamoxifen in the in prevention of breast cancer for postmenopausal women on HRT (37). Postmenopausal women on HRT (n= 1884), were randomly assigned to either tamoxifen or placebo. The dose of tamoxifen used was lower than the other trials (5 mg/day compared to 20 mg/day in prior trials). At follow up, 24 women were diagnosed with breast cancer on placebo compared to 19 on tamoxifen, but the results were not statistically significant (RR 0.80, 95% CI 0.44 to 1.46). Cardiovascular events, cerebrovascular events, thrombotic events, and uterine cancer events did not differ between the two groups.

More recently, in Italian investigators updated their TAM-01 study (38), which randomly assigned 500 women with breast intraepithelial neoplasm (IEN), including atypical ductal hyperplasia (20%), LCIS (11%), or hormone-sensitive DCIS (69%), after surgery, to mini-dose tamoxifen (5 mg once daily) or placebo. Participants were mostly postmenopausal (58%). After a median follow-up of 9.7 years, 25 breast cancers were diagnosed in the tamoxifen group compared to 41 in the placebo group (annual rate per 1,000 person-years, 11.3 with tamoxifen versus 19.5 with placebo; hazard ratio [HR], 0.58; 95% CI, 0.35 to 0.95; log-rank p= 0.03). Contralateral breast cancers were also lower in the mini-tamoxifen group (6 events) compared to 16 in the placebo arm (HR, 0.36; 95% CI, 0.14 to 0.92; p= 0.025). The number needed to be treated to prevent one case of breast event with mini-tamoxifen was 22 in 5 years and 14 in 10 years. No difference in the incidence of serious adverse events was reported in the two groups during the prolonged follow-up period (39).

The United States Preventive Services Task Force (USPSTF) included four trials that used tamoxifen for breast cancer prevention in their meta-analysis (n=28421). The meta-analysis revealed that administration of tamoxifen over five years leads to a reduction in the risk of breast cancer at twenty years. The risk of invasive breast cancer cases dropped to 7 per 1000 women over five years (95% CI: 0.59 to 0.84). The incidence of ER-positive breast cancer exhibited a greater reduction compared to ER-negative breast cancer. Additionally, Tamoxifen yielded superior results in women who were not on HRT compared to when tamoxifen was used in conjunction HRT for menopause (31).

While tamoxifen has demonstrated efficacy in reducing the risk of breast cancer, it is not without side effects. In the updated USPSTF meta-analysis, tamoxifen was associated with increased incidence of endometrial cancer (RR 2.25, 95% CI, 1.17 to 4.41). Tamoxifen was also linked to an increase in thromboembolic events. Risks for thromboembolic events and endometrial cancer with tamoxifen were higher for older compared with younger women and returned to normal after discontinuation in two trials included in the meta-analysis (RR 0.98, 95% CI 0.48 to 1.80) (31). In some studies, tamoxifen has been associated with a modest increase in bone density, which might have a protective effect against osteoporosis. However, in other cases, especially in postmenopausal women, tamoxifen use has been linked to a potential acceleration of bone loss and increasing the risk of osteoporosis.

A few studies also examined the impact of tamoxifen on the quality of life (QoL) in postmenopausal women at high risk of breast cancer. The Study of Tamoxifen and Raloxifene (STAR) enrolled more than 19,000 high-risk postmenopausal women. Patient-reported symptoms were collected using a 36-item checklist, while QoL was assessed through three different questionnaires (40). Women in the tamoxifen group reported greater mean symptom severity for gynecological problems (0.29 vs 0.19, p < 0.001), vasomotor symptoms (0.96 vs 0.85, p < 0.001), leg cramps (1.10 vs 0.91, p < 0.001), and bladder control symptoms (0.88 vs 0.73, p < 0.001). Another clinical trial randomized 235 premenopausal women at higher risk to tamoxifen 5 mg/day, fenretinide 200 mg/day, their combination, or placebo (41). The study showed no statistically significant difference among the four treatment arms for all four domains (vasomotor, physical, psychosocial, and sexual). Additional studies did not demonstrate a clear link between taking tamoxifen and major psychosocial outcomes like depression, anxiety, or overall quality of life (42, 43).

### 3.3 Raloxifene

Raloxifene, a selective SERM, is used for the treatment and prevention of osteoporosis in postmenopausal women (44). Unlike tamoxifen, raloxifene exhibits antiestrogenic effects on the endometrium and thus may not be associated with endometrial cancer. Raloxifene has been evaluated in multiple studies to test its ability to prevent breast cancer. The MORE (Multiple Outcomes of Raloxifene Evaluation) trial was a multicenter, randomized, double-blind study designed to assess bone fracture risk and breast cancer risk with three years of raloxifene use (45). The trial included 7705 postmenopausal women with a history of osteoporosis, and participants were randomized into three groups: raloxifene at 120 mg/day, raloxifene at 60 mg/day, or a placebo. The primary endpoint was the impact of raloxifene on bone health, and it also prospectively assessed whether raloxifene could reduce the risk of breast cancer (secondary endpoint). The median age of participants was 66.5 years, and they were mainly of European ancestry. Only 12.3% of the women reported a family history of breast cancer. With a median follow-up of 40 months, raloxifene significantly reduced the incidence of breast cancer in the group receiving it compared to placebo. The relative risk for invasive breast cancer in the raloxifene group was 0.24 (95% CI 0.13 to 0.44, p < 0.001), and the reduction in risk was similar for both doses of raloxifene. The benefits were most significant for estrogen receptor (ER)-positive breast cancers, with a relative risk of 0.10 (95% CI 0.04 to 0.24). Raloxifene did not significantly change the risk of ER-negative breast cancer. Side effects included more hot flashes, leg cramps, and peripheral edema in the raloxifene group. Importantly, there was no increase in vaginal bleeding or endometrial cancers. However, raloxifene increased the risk of thromboembolic events (RR 3.1 95% CI 1.5 to 6.2) (46, 47).

As an extension of the MORE trial, the CORE clinical trial was a multicenter, placebo-controlled study that aimed to investigate the effect of an additional 4 years of raloxifene (at 60 mg/day) on the incidence of invasive breast cancer in postmenopausal women with osteoporosis (48). Out of 6511 participants from 180 sites in 24 countries in the MORE trial, 4011 enrolled in the CORE trial. The primary objective was the incidence of invasive breast cancer, with a secondary objective focused on ER-positive breast cancer. Results showed that women in the raloxifene group had a 59% reduction in the incidence of invasive breast cancer compared with the placebo group [2.1 vs. 5.2 cases per 1000 woman-years (HR 0.41, 95% CI 0.24 to 0.71; p < 0.001)]. The reduction was even more significant for ER-positive breast cancers, with a 66% reduction (HR 0.34, 95% CI 0.18 to 0.66; p < 0.001). Overall, the incidence of breast cancer, regardless of invasiveness, was reduced by 50% in the raloxifene group compared with placebo. Adverse events were reported in 80% of patients, with no statistically significant differences between the raloxifene and placebo groups. The incidence rates for vaginal bleeding, endometrial hyperplasia, endometrial cancer, hot flashes, leg cramps, and peripheral edema were not significantly different between raloxifene and placebo groups during the 4-year CORE trial (p > 0.05 for each event). However, hot flashes and leg cramps were reported more often in the raloxifene group over the 8-year period (p < 0.001 and p= 0.008, respectively). The incidence of thromboembolic events was higher in the raloxifene group, but the difference was not statistically significant during both trials. The findings from the CORE study suggest that the benefits of raloxifene endure over time for invasive breast cancer, even though its impact on noninvasive diseases is comparatively limited.

The RUTH (Raloxifene Use for The Heart) trial involved more than 10,000 postmenopausal women either with established coronary heart disease (CHD) or at an increased risk for CHD. Participants were randomly assigned to receive 60 mg of raloxifene daily or placebo. The trial had a median follow-up duration of 5.56 years and assessed coronary events, breast cancer, stroke, venous thromboembolism (VTE), and death. Raloxifene significantly reduced the incidence of invasive breast cancer (hazard ratio, 0.56; 95% CI, 0.38 to 0.83; p= 0.03), particularly ER-positive cases, with notable absolute risk reductions. Raloxifene use did not significantly impact the primary outcome of death from coronary causes, nonfatal myocardial infarction, or hospitalization for acute coronary syndromes compared to the placebo group (HR, 0.95; 95% CI, 0.84 to 1.07; p > 0.05). The use of cardioprotective medications did not differ significantly between the treatment groups. Adverse events were comparable between groups, with some differences in specific side effects. Secondary outcomes revealed a higher incidence of fatal stroke and venous thromboembolic events in the raloxifene group. The study found lower rates of clinical vertebral fractures (HR, 0.65; 95% CI 0.47 to 0.89, ARR 1.3 per 1000), but no significant reduction in nonvertebral fractures in the raloxifene group, which was consistent with results from the MORE (49).

These major trials were also included in the outcomes from the USPSTF updated meta-analysis for invasive breast cancer risk reduction, with pooled risk ratio of 0.44 (95% CI, 0.24 to 0.80). Raloxifene was associated with reduced ER-positive but not ER-negative invasive breast cancer. Raloxifene was also associated with a reduced risk of vertebral fractures (7 cases in 1000 women: RR 0.61, 95% CI 0.53 to 0.73). Pooled data from the MORE/CORE, and RUTH trials were associated with increased thromboembolic events (RR, 1.56, 95% CI, 1.11 to 2.60). The meta-analysis showed that the use of Raloxifene did not increase the occurrence of endometrial cancer (RR, 0.55, 95% CI, 0.36 to 0.83), or endometrial hyperplasia (RR, 0.19, 95% CI, 0.12 to 0.29).

### 3.4 Tamoxifen vs. Raloxifene

The Study of Tamoxifen and Raloxifene (STAR) trial involved more than 19,000 postmenopausal women aged 35 or older with a history of lobular carcinoma *in situ* (LCIS) treated by local excision alone or a modified Gail score indicating a 5-year risk for invasive breast cancer of at least 1.66% (17). The trial aimed to compare the efficacy and safety of tamoxifen (20 mg/d) and raloxifene (60 mg/d) over 5 years follow up. The study found no significant difference in the incidence of invasive breast cancer between tamoxifen and raloxifene groups in all subgroups (RR 1.02, 95% CI, 0.82 to 1.28, p= 0.96). However, fewer cases of non-invasive breast cancer were reported in the tamoxifen group, but the difference was not statistically significant (RR 1.40, 95% CI, 0.98 to 2.00, p= 0.052). Endometrial cancer incidence was 38% lower in the raloxifene group than in the tamoxifen group, although not statistically significant. However, patients in the raloxifene group had significant reduction in the number of hysterectomies (RR 0.44; 95% CI 0.35 to 0.56), as well as reduction endometrial hyperplasia (RR 0.16; 95% CI 0.09 to 0.29). Raloxifene also demonstrated significant reduction in the risk of thromboembolic events (RR, 0.70; 95% CI, 0.54 to0.91) compared to tamoxifen, with 36% reduction in pulmonary embolism (PE) and 26% reduction in deep vein thrombosis (DVT). Cataract incidence and cataract surgery were less frequent in the raloxifene group (RR, 0.79; 95% CI, 0.68 to0.92 and RR, 0.82; 95% CI, 0.68 to0.99, respectively). Overall, both treatments exhibited similar mortality rates and did not significantly differ in the incidence of ischemic heart disease or fractures. Together, these findings indicate that raloxifene serves as an alternative to tamoxifen for reducing the risk of invasive breast cancer in postmenopausal women with elevated Gail risk scores and in individuals with LCIS.

Cuzick et al. updated available data published before 2003 for tamoxifen and raloxifene and provided an overview of the combined results (15). Data from IBIS-I, National NSABP-P1, Italian trial, the Royal Marsden Hospital trial, and the MORE trial were included. The combined results on tamoxifen’s impact on breast cancer incidence showed a substantial reduction of 30 to 40% with tamoxifen (95% CI 28 to 46, p < 0.0001). The MORE trial demonstrated an even more significant reduction of 64% (44 to 78%, p < 0·0001). ER-negative breast cancers did not exhibit a reduction, while ER-positive cancers were significantly reduced by 48% (36 to 58%, p < 0.0001). Tamoxifen increased the risk of endometrial cancer (RR 2.25, 1.17 to 4.41, p= 0.0005). Venous thromboembolic events were elevated with tamoxifen (RR 1.9, 1.4 to 2.6, p < 0.0001), and varying death rates were noted across studies, with no significant impact on all-cause mortality in tamoxifen prevention trials (HR 0.90, 0.70 to 1.17, p= 0.44).

### 3.5 Aromatase inhibitors

The AIs inhibit the enzyme aromatase, which is responsible for the peripheral conversion of androgens to estrogens. When used in the treatment of early-stage breast cancer in the adjuvant setting, anastrozole was associated with 50% relative reduction in risk of developing contralateral breast cancer (50).

Goss et al. studied the effect of exemestane in reduction of breast cancer in the Mammary Prevention3 (MAP.3) trial evaluating exemestane for breast cancer risk reduction (16). The trial included 4,560 postmenopausal women who met at least one of the following risk factors: age ≥ 60 years, Gail risk score >1.66%, history of atypical ductal or lobular hyperplasia, lobular carcinoma *in situ*, or ductal carcinoma *in situ* treated with mastectomy. At a median follow-up of 35 months, exemestane was shown to decrease invasive breast cancers in all study subgroups (HR 0.35, 95% CI 0.18 to 0.70). The number needed to treat to prevent one case of invasive breast cancer was 94 in 3 years and 26 in 5 years. Adverse events occurred in 88% of women in the exemestane group and 85% in the placebo group (p= 0.003). Arthritis and hot flashes were more common in the exemestane group, but differences in grade 2 or higher symptoms were modest. No significant differences were observed in osteoporosis, cardiovascular events, or clinical fractures. There were 38 deaths during the study, with no significant differences in causes of death between the two groups. Despite self-reported worsening of menopause-related vasomotor and sexual symptoms, the study reported no significant differences in QoL responses.

The International Breast Cancer Intervention Study II (IBIS-II) was a randomized, placebo-controlled study that investigated the impact of anastrozole on invasive breast cancer in postmenopausal women who were randomly assigned to receive anastrozole at 1 mg/day or placebo daily (51). The study included 4,560 postmenopausal women with a median age of 59 years. Significant reductions in breast cancers, including ductal carcinoma *in situ* (HR 0.47, 95% CI 0.32 to 0.68, p < 0.0001), were observed in the anastrozole group over a median follow-up of five years. Anastrozole additionally showed a more pronounced effect on reducing high-grade tumors, particularly in estrogen-receptor-positive, progesterone-receptor-positive, and node-negative tumors. No significant heterogeneity was found in different subgroups, although larger effects were noted for women with lobular carcinoma *in situ* or atypical hyperplasia. Overall mortality did not significantly differ between the groups, with 18 deaths reported in the anastrozole group compared to 17 in the placebo group. While the total number of fractures did not differ between groups, musculoskeletal adverse events were significantly more common with anastrozole. The anastrozole group reported more moderate arthralgia (RR 1.10, 95% CI 1.05 to 1.16), carpal tunnel syndrome (RR 0.23, 95% CI 0.99 to 1.51), and vasomotor symptoms (RR 1.15, 95% CI 1.08 to 1.22). Hypertension increased significantly with anastrozole (RR 1.64, 95% CI 1.18 to 2.28), but no significant differences were noted in thromboembolic events, cerebrovascular events, or myocardial infarction frequencies.

A recently published case-control study using data from the IBIS-II trial examined how baseline blood hormone levels affected anastrozole’s preventive efficacy in high-risk postmenopausal women (52). The analysis focused on estradiol, testosterone, and sex hormone-binding globulin (SHBG) concentrations. Anastrozole demonstrated a clear benefit in reducing breast cancer risk across all quartiles of estradiol–SHBG ratio. In contrast, the placebo group showed an increasing risk with higher quartiles of estradiol–SHBG ratio (trend per quartile 1.25, 95% CI 1.08 to 1.45, p= 0.0033). The testosterone–SHBG ratio also exhibited a significant association with breast cancer risk in the placebo group (trend 1.21, 95% CI 1.05 to 1.41, p= 0.011) but not in the anastrozole group.

**Figure 1:**
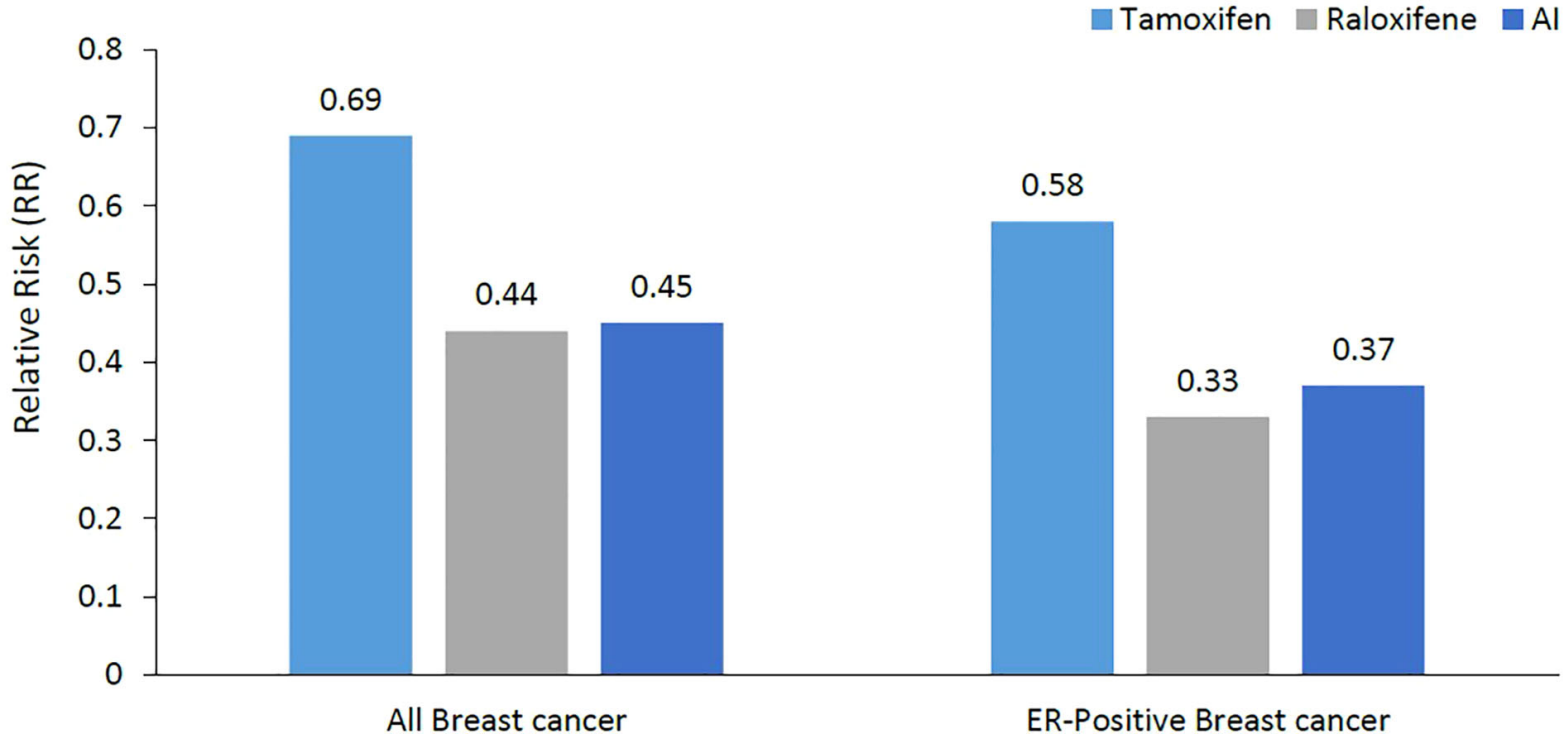
Relative risk reduction of breast cancer (all breast cancers and ER-positive breast cancer). Much of the breast cancer risk reduction benefit was in the ER-positive subtype. ER, Estrogen Receptors; AI, Aromatase Inhibitors.

**Figure 2:**
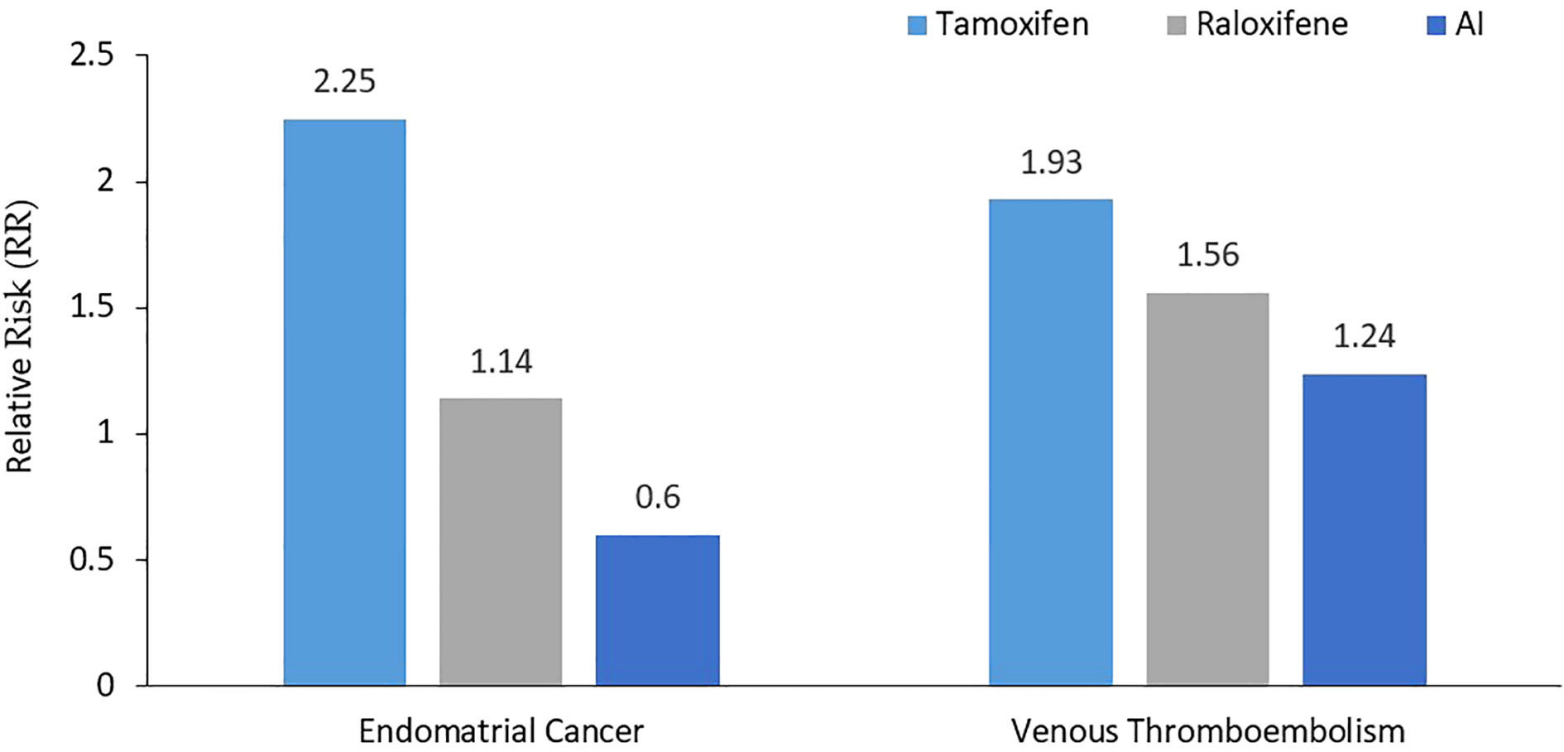
Relative risk of both endometrial cancer and venous thromboembolism. Compared to tamoxifen, AI were associated with lower risk of both endometrial cancer and VTE, while raloxifene was not worse than tamoxifen for VTE, but was associated with lower risk of endometrial cancer. AI, Aromatase Inhibitors.

The MAP.3 trial and the IBIS-II trial were also included in the outcomes from the USPSTF updated meta-analysis for invasive breast cancer risk reduction, with pooled risk ratio of 0.45 (95% CI, 0.26 to 0.70) (31). Both trials were associated with reduced estrogen receptor–positive but not estrogen receptor–negative invasive breast cancer. Unlike tamoxifen and raloxifene, AI had no effect on fractures in the meta-analyses. **Figure 1** summarizes the breast cancer prevention benefits associated with tamoxifen, raloxifene and AI, while the risk of endometrial cancer and VTE are shown in **Figure 2**.

### 3.6 Chemoprevention for inherited breast cancer

Women with pathogenic *BRCA1* or *BRCA2* variants have many options to reduce their known high risk of developing breast cancer (53). Obviously, risk reducing surgeries; mastectomy and bilateral salpingo-oophorectomy BSO), are well studied and many women had gone through this pathway, on what’s is known now in the literature as “Angelina Jolie effect”. Data on the utilization of tamoxifen remain scarce (54–56) and mostly related to the NSABP-P1 trial in which 288 women develop breast cancer; 8 had *BRCA1* and 11 had *BRCA2* (54).

Aromatase inhibitors were initially evaluated in secondary prevention. In one study from MDACC and Baylor College, 935 patients diagnosed with early-stage ER+ breast cancer with known BRCA mutation status between 2004 and 2014 were followed for a median time of 11.5 years. Among the subjects included in the analysis, 53 had *BRCA1* and 94 had *BRCA2*. A total of 66 contralateral breast cancers (CBCs) were reported; 51(6.5%) of the 788 women without BRCA compared to 15 (10%) of the 147 women with pathogenic *BRCA1*/2 variants. Women who used AI had a significant reduction in risk of CBC (HR 0.44, p = 0.004), regardless of the BRCA mutation status while tamoxifen use was not associated with any beneficial effect (57). Another trial, The French LIBER Trial, is investigating the effect of letrozole at a dose of 2.5 mg daily for 5 years in the primary prevention of breast cancer among unaffected 171 postmenopausal women aged 40–70 (58).

BRCA-P trial is an ongoing randomized study evaluating the preventive effect of denosumab in healthy *BRCA1* germ line mutation carriers. The study is based on the hypothesis that women with BRCA pathogenic variants have high estrogen level and their mammary stem cells lacks sex hormone binding globin, and these stem cells are stimulated with a mechanism similar to that of osteoclast utilizing RANK/RANKL (59). It is hoped that denosumab would interfere with the RANKL/RANK-stem cell stimulation and thus prevent breast cancer.

### 3.7 Uptake of chemoprevention

Limited awareness, concerns about side effects associated with tamoxifen and AIs, and the limitations of breast cancer risk prediction models contribute to the poor uptake of chemoprevention (60). In one study from Leeds, 1620 women with moderate- or high-risk family history of breast cancer were counselled about chemoprevention in breast clinics, following which questionnaire survey was subsequently sent to these patients to explore factors that may influence their willingness to start chemoprevention. One-third (n = 518, 32%) responded, and only 56 (10.8%) agreed to start chemoprevention. The most common barriers were side effects (79.4%) and lack of information (53%) (61).

Researchers are exploring new strategies that may potentially reduce adverse events while preserving the beneficial anticancer properties of chemoprevention drugs. Such strategies include low-dose tamoxifen (5 mg), and the utilization of biomarkers, or postmenopausal hormonal level to better select women who would benefit the most. Transdermal drug delivery using gels was also investigated as a potentially effective alternative to oral tamoxifen or AI (62, 63). Recent studies have shown that pharmacogenomic variants have the potential to guide personalized endocrine treatment (64)

## 4 Conclusions

Breast cancer remains a global health challenge. Risk factors, including age, family history, lifestyle choices, and genetic predispositions, contribute to the intricate etiology of breast cancer, emphasizing the necessity for personalized risk assessment tools. Chemoprevention, particularly with agents like tamoxifen, raloxifene, and aromatase inhibitors, has been studied for reducing breast cancer risk. Randomized controlled trials have demonstrated the ability of chemoprevention to significantly lower the risk of breast cancer. However, the choice of chemopreventive agents requires consideration of individual risk profile, potential side effects, and patients’ preference. A comprehensive and individualized approach to risk assessment and chemoprevention is crucial for effectively addressing the multifaceted challenge of breast cancer prevention.
